# Better outcome for parotid *versus* neck metastasis of head and neck cutaneous squamous cell carcinoma: a new report on reemerging data

**DOI:** 10.1016/j.bjorl.2019.10.007

**Published:** 2019-11-15

**Authors:** Fábio Muradás Girardi, Vivian Petersen Wagner, Manoela Domingues Martins, Aliende Lengler Abentroth, Luiz Alberto Hauth

**Affiliations:** a Centro de Oncologia Integrada do Hospital Ana Nery, Santa Cruz do Sul, RS, Brazil; b Universidade de Campinas, Faculdade de Odontologia de Piracicaba, Departamento de Diagnóstico Oral, Piracicaba, SP, Brazil; c Universidade Federal do Rio Grande do Sul, Faculdade de Odontologia, Departamento de Patologia Oral, Porto Alegre, RS, Brazil; d Universidade Federal do Rio Grande do Sul, Hospital das Clínicas de Porto Alegre, Departamento de Medicina Oral, Porto Alegre, RS, Brazil

**Keywords:** Carcinoma squamous cell, Skin neoplasms, Lymphatic metastasis, Prognosis, Treatment outcome

## Abstract

**Introduction:**

Regional metastases of cutaneous head and neck squamous cell carcinoma occur in approximately 5 % of cases, being the most important prognostic factor in survival, currently with no distinction between parotid and neck metastasis.

**Objective:**

The purpose of this study was to evaluate the prognostic features among patients with head and neck cutaneous squamous cell carcinoma exhibiting regional metastasis.

**Methods:**

A retrospective analysis of patients with cutaneous squamous cell carcinoma who underwent parotidectomy and/or neck dissection from 2011 to 2018 at a single institution tertiary center was performed. Patient demographics, clinical, surgical and pathological information, adjuvant treatments, and outcome at last follow-up were collected. Outcomes included disease recurrence and death due to the disease. Prognostic value of clinic pathological features associated with disease-specific survival was obtained.

**Results:**

Thirty-eight cases of head and neck cutaneous squamous cell carcinoma with parotid and/or neck metastasis were identified. Overall, 18 (47.3 %) patients showed parotid metastasis alone, 12 (31.5 %) exhibited neck metastasis alone and 8 (21.0 %) had both. A primary tumor in the parotid zone (Hazard Ratio ‒ HR = 5.53; *p* = 0.02) was associated with improved disease-specific survival. Poorer disease-specific survival was observed in patients with higher primary tumor diameter (HR = 1.54; *p* = 0.002), higher depth of invasion (HR = 2.89; *p* = 0.02), invasion beyond the subcutaneous fat (HR = 5.05; *p* = 0.002), neck metastasis at first presentation (HR = 8.74; *p* < 0.001), number of positive lymph nodes (HR = 1.25; *p* = 0.004), and higher TNM stages (HR = 7.13; *p* = 0.009). Patients presenting with isolated parotid metastasis during all follow-ups had better disease-specific survival than those with neck metastasis or both (HR = 3.12; *p* = 0.02).

**Conclusion:**

Head and neck cutaneous squamous cell carcinoma with parotid lymph node metastasis demonstrated better outcomes than cases with neck metastasis.

## Introduction

According to the latest GLOBOCAN report, non-melanoma skin cancer (excluding basal cell carcinoma) is the fifth most common malignant tumor in the world.^1^ Moreover, in the next 20 years, an increase of 90.2 % is expected, leading to an incidence of almost 2 million people by 2040.^2^ Cutaneous squamous cell carcinoma (CSCC) is the most common form of non-melanoma skin cancer, when basal cell carcinoma is excluded. The head and neck regions are affected in more than 80 % of cases. Fortunately, the prognosis is usually very good, and the low percentage of deaths occurs mainly due to metastatic disease. Regional metastases of CSCC occur in approximately 5 % of cases^3^ and malignant cells have the potential to metastasize to intraglandular parotid and/or neck lymph nodes.^4^ Besides directly affecting the mortality of CSCC, the presence of regional metastasis has an important effect on morbidity, once patients might require multimodality treatment such as neck dissection, different extents of parotidectomy (with or without facial nerve preservation), and postoperative radiation therapy.^5^

Staging systems represent a pivotal tool for prognostic stratification enabling physicians to plan treatment based on tumor risk.^6^ Currently, the American Joint Committee on Cancer (AJCC) staging manual represents the reference guideline for classifying patients with different types of cancer, including CSCC. This manual is continually revised to incorporate important features that might affect the prognosis on high evidence-based levels. Since its first release in 1997, eight editions have been produced. In the seventh edition, important features such as depth of invasion, perineural invasion, and histologic grade were included for the first time.^7^ Launched in 2017, the eighth edition has incorporated different and important risk factors based on cohort studies that emerged since the seventh edition, such as large caliber or subdermal perineural invasion, minor bone erosion, and invasion beyond subcutaneous tissue or depth of invasion (DOI) > 6 mm. Concerning the nodal metastasis (N) category, the presence of Extranodal Extension (ECE) must now be taken into account and denotes a more advanced stage.^8^ Recently, Moeckelmann et al. evaluated the performance of the AJCC 8 nodal staging system in a retrospective Australian cohort of 382 head and neck CSCC cases. The current nodal staging system did not provide any risk stratification, suggesting CSCC merits an independent nodal staging system.^9^

In 2002, O’Brien et al. published the first report demonstrating better prognosis of parotid involvement compared with neck disease.^10^ Since then, several reports have been conducted in this subject and heterogeneous findings obtained.^11^, ^12^, ^13^, ^14^, ^15^ The current TNM staging system considers that although preliminary data suggest that cervical nodal disease may portend a worse prognosis than parotid disease, the data are insufficient to support this separation yet.^16^ The purpose of this study was to revisit this topic and evaluate the prognostic features among patients with head and neck CSCC regional metastasis.

## Methods

### Study population

A retrospective analysis was performed at a single tertiary center in southern Brazil (Integrated Oncology Center of Ana Nery Hospital - Santa Cruz do Sul, Brazil) between January 1, 2011, and December 31, 2018. Patients were identified from the electronic medical record using the 10th revision of the International Classification of Diseases (ICD-10) codes (C07/C77.0 and C44). Pathological reports of all head and neck surgeons from the service were also reviewed, searching for parotidectomy and/or neck dissection and previous history of head and neck skin cancer. Patients without a history of head and neck CSCC, those with previous SCC from the upper aerodigestive tract and those whose surgery was performed at another center were excluded. Information gathered included patient demographics, clinical data, surgical and pathological information, adjuvant treatments, and outcome at last follow-up were recorded. Outcomes included disease recurrence and death due to the disease. Patients who died for other causes were treated as censored cases based on the death date. All patients were kept on follow-up at our institution. Survival time was calculated as the interval from surgery for the primary tumor to the date of death or last contact. The median follow-up period was calculated including only patients alive at the end of the study. This study was conducted after approval by the local ethics committee (CAAE: 93792318.4.0000.5304).

### Statistical analysis

Data were analyzed using SPSS software (IBM Corporation, Armonk, NY), version 20.0. Initially, a descriptive analysis of clinic pathological features was performed. The univariate Cox proportional hazard regression model was used to evaluate the prognostic value of clinic pathological features associated with disease-specific survival (DSS). The assumption of proportional hazards was verified for all variables evaluated. Kaplan–Meier cumulative DSS curves were generated and compared using the log-rank test. Spearman’s correlation test was used to determine the correlation of survival time and period between initial surgery and metastasis (only for patients who died due to the disease). For all tests, *p* ≤ 0.05 was considered indicative of statistical significance.

## Results

### Demographics and clinicopathological features

Thirty-eight cases of head and neck CSCC with parotid and/or neck metastasis were identified. All cases were selected for analysis. Relevant demographic and clinicopathological data are summarized in Table 1.

**Table 1:** Demographic and clinicopathological features of patients with metastatic cutaneous squamous cell carcinoma.

	n = 38
Gender	
Male	24 (63.2%)
Female	14 (36.8%)
Age at diagnosis	
Mean (±SD)	74.98 (±12.42)
Range	38– 91
Site	
Parotid zone	11 (28.9%)
Others	27 (71.1%)
Clark level	
III	2 (5.3%)
IV	12 (31.6%)
V	22 (57.9%)
Missing	2 (5.3%)
Tumor diameter (cm)	
Mean (±SD)	3.32 (±1.75)
Range	0.80–8.00
Invasion beyond adipose tissue	
Yes	17 (44.7%)
No	19 (55.0%)
Missing	2 (5.3%)
Surgical margins (primary tumor)	
Positive	8 (21.1%)
Close	7 (18.4%)
Negative	22 (57.9%)
Perineural invasion	
Absent	24 (63.2%)
Present	13 (34.2%)
Missing	1 (2.6%)
Angiovascular invasion	
Absent	18 (47.4%)
Present	19 (50.0%)
Missing	1 (2.6%)
Differentiation grade	
I	13 (34.2%)
II	18 (47.4%)
III	6 (15.8%)
Missing	1 (2.6%)
DOI (cm)	
Mean (±SD)	1.37 (0.84)
Range	0.30–2.70
TNM (8th edition)	
I	5 (13.2%)
II	7 (18.4%)
III	16 (46.1%)
IV	9 (23.7%)
Missing	1 (2.6%)
Metastasis at initial presentation	
Absent	27 (71.1%)
Neck	3 (7.9%)
Parotid gland	7 (18.4%)
Missing	1 (2.6%)
Site of metastasis during follow	
Parotid gland	18 (47.4%)
Parotid gland and neck	8 (21.1%)
Neck	12 (31.6%)
Number of positive LN	
Mean (±SD)	2.30 (±2.17)
Range	1–10
ECE	
No	2 (5.3%)
Yes	29 (76.3%)
Missing	7 (18.4%)
Outcome	
Alive with disease	4 (10.5%)
Alive free of disease	14 (36.8%)
Death due to the tumor	18 (47.3%)
Death for other reasons	2 (5.3%)

SD, Standard Deviation; DOI, Depth of Invasion; LN, Lymph Node; ECE, Extracapsular Extension.

The mean age was 74.8 years (range 38–91 years). There was male/female preponderance (63.2 %). Immunosuppression was documented in two patients (5.2 %). The median primary head and neck CSCC tumor diameter was 30 mm (range 8–80 mm), removed mainly from the parotid zone (28.9 %). Other sites of the primary tumor are listed in Table 1. Most of the primary tumors (21; 55.2 %) were classified as T3 and moderately differentiated histopathological grade (47.3 %). The median time from primary surgery to lymph node/parotid dissection was 3.7 months (range 0–44.1 months). At initial evaluation, only 10 patients (26.3 %) presented metastasis, six of them presenting recurrences during follow-up. The other 28 cases presented metastasis only during the follow-up. Overall, 18 (47.3 %) patients had parotid metastasis alone, 12 (31.5 %) patients exhibited neck metastasis alone and 8 (21.0 %) had both. The median number of positive lymph nodes dissected from the neck and parotid was 1 (range 1–10). Twenty cases presented with one single metastasis, fifteen of them on parotid, and five of them died of the disease. Among the five cases with single neck metastasis, there were two deaths by disease. Twenty-nine cases (76.3 %) of the involved nodal specimens demonstrated ECE.

### Treatment modalities

Surgery was the definitive mode of treatment in 36 of the 38 patients (94.7 %), whereas the other 2 patients received radiotherapy alone. Overall, combined parotidectomy and neck dissection was carried out in 25 (65.7 %) of the surgically treated patients, whereas 6 (15.7 %) patients had neck dissection only and 7 (18.4 %) had parotidectomy only. Thirty-three patients (86.8 %) received adjuvant external-beam radiation treatment (78.7 % completion rate). The main reason for avoiding radiation was patient being unfit to receive adjuvant radiation (n = 3; 7.8 %), followed by patient refusal (n = 2; 5.2 %). The median radiation dose was 60 Grays (range 8–66 Grays) delivered over a median of 30 fractions. Five (13.1 %) were submitted to adjuvant radiation combined with chemotherapy (cisplatin) after surgery.

### Survival analysis

**Figure 1:**
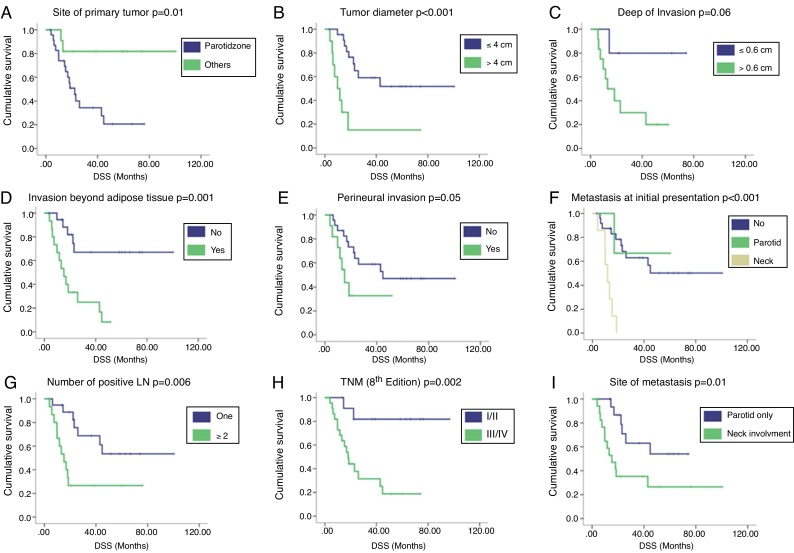
Kaplan–Meier cumulative survival curves according to (A) site of primary tumor, (B) tumor diameter (cm), (C) depth of invasion (cm), (D) invasion beyond adipose tissue, (E) perineural invasion, (F) presence of metastasis at initial presentation, (G) number of positive lymph nodes, (H) clinical stage according to the 8^th^ edition of AJCC, and (I) site of metastasis. Curves were compared using the log-rank test. DSS, Disease Specific Survival; LN, Lymph Node.

With a median follow-up of 51.7 months, the 2 year and 5 year DSS rates were 39.2 % and 25.9 %, respectively. Regional recurrences occurred in 33 (86.8 %) patients, 30 (90.9 %) of them during the first 2 years after primary surgery. Univariate Analysis (UVA) of predictors of DSS is described in Table 2. The primary tumor on the parotid zone (HR = 5.53; *p* = 0.02) was associated with improved DSS on UVA. Poorer DSS was observed in patients with higher primary tumor diameter (HR = 1.54; *p* = 0.002), higher DOI (HR = 2.89; *p* = 0.02), invasion beyond the subcutaneous fat (HR = 5.05; *p* = 0.002), neck metastasis at first presentation (HR = 8.74; *p* < 0.001), number of positive lymph nodes (HR = 1.25; *p* = 0.004), and higher TNM stages (HR = 7.13; *p* = 0.009). Patients presenting with isolated parotid metastasis during all follow-ups had better DSS than those with neck metastasis or both (HR = 3.12; *p* = 0.02). A correlation between the time from primary surgery to regional metastasis and DSS was also identified (correlation coefficient = 0.782; *p* = 0.05). The Kaplan–Meier cumulative survival curves according to DSS predictors are presented in Fig. 1. Curves were compared using the log-rank test.

**Table 2:** Cox univariate analysis of patient and tumor characteristics on survival.

Variable	HR (95% CI)	*p*-value
Gender		
Female	1	
Male	1.11 (0.41–2.97)	0.83
Age	1.01 (0.97–1.05)	0.45
Site		
Parotid zone	1	
Others	5.53 (1.26–24.28)	0.02
Differentiation grade		
I	1	
II	1.09 (0.39–3.01)	0.86
III	0.52 (0.10–2.57)	0.42
Tumor Diameter (cm)	1.54 (1.16–2.03)	0.002
DOI (cm)	2.89 (1.13–7.37)	0.02
Invasion beyond adipose tissue		
No	1	
Yes	5.05 (1.78–14.29)	0.002
Clark level		
III/IV	1	
V	2.46 (0.87–6.93)	0.87
Surgical margins (primary tumor)		
Negative	1	
Positive/Close	1.87 (0.74–4.74)	0.18
Perineural invasion		
Absent	1	
Present	2.58 (0.96–6.97)	0.06
Angiovascular invasion		
Absent	1	
Present	1.82 (0.71–4.65)	0.20
Metastasis at initial presentation		
No	1	
Parotid	0.88 (0.11–6.91)	0.90
Neck	8.74 (2.75–28.03)	<0.001
Number of positive LN	1.25 (1.07–1.46)	0.004
TNM 8th Edition		
I/II	1	
III/IV	7.13 (1.62–31.25)	0.009
Site metastasis		
Parotid only	1	
Neck involvment	3.12 (1.16–8.36)	0.02

HR, Hazard Ratio; CI, Confidence Interval; DOI, Depth of Invasion; LN, Lymph Node.

## Discussion

The fast evolution of knowledge in cancer biology imposes the necessity of continuous questioning and updates in staging systems aiming to supply cancer care providers the best evidence-based resource for classifying patients, defining prognosis, and determining the most appropriate treatment protocol. The AJCC Eighth Edition Team made important efforts to build a more “personalized” approach for cancer staging. Recent evidence has already demonstrated that the new proposed system for CSCC has improved homogeneity and monotonicity besides having a better prognostic value.^17^ In spite of that, an Australian group recently demonstrated that the current nodal staging system does not add any prognostic information, suggesting that an independent nodal staging system would be more appropriate concerning head and neck CSCC.^9^ Our clinical impression led us to question whether the site of node involvement should not be taken into consideration for the analysis of the N category. Here, we demonstrated a better prognosis for cases with parotid *versus* neck metastasis in our cohort of patients. This finding might reignite the debate of the possibility to incorporate this feature in a future staging system.

In 2002, O’Brien et al.^10^ hypothesized that, regarding head and neck CSCC, better prognostic discrimination might be achieved by dividing parotid and neck disease. Analyzing a sample of 87 cases, the authors found that, among patients with metastatic CSCC involving the parotid gland, those who also had the disease in the neck had a statistically significantly worse outcome.^10^ A following multicenter study was conducted on 322 patients from three Australian and three North American institutions in 2006, confirming initial impressions that the addition of parotid and neck stages adds valuable prognostic information about cancer-specific survival.^11^ Ch’ng et al.^12^ revisited this issue, showing worse prognosis according to parotid or neck progression of the disease, and impact on outcomes of cumulative parotid and neck metastasis compared with single site disease. Nevertheless, conflicting subsequent results^13^, ^14^, ^15^ made the current AJCC staging system team consider that there were insufficient data to support a separation of parotid and neck disease at this time.^16^ Probably the most impacting results are from Hirshoren et al. that studied 183 cases of metastatic head and neck CSCC. They found no association between overall survival and site of metastasis (parotid *versus* neck *versus* both), but only with lymph node ratio.^13^ Those results followed a trend in head and neck oncology, joining with several other publications regarding different head and neck topographies.^18^

According to the AJCC, current and future changes to AJCC staging content must rely on evidence and the highest cancer level of evidence comprehends “consistent results from multiple large, well-designed, and well-conducted national and international studies in appropriate patient populations, with appropriate endpoints and appropriate treatments”. To our knowledge, this is the first study to address the role of site of metastasis as a prognostic marker in a South American population. We believe that this is important because it might increase the level of evidence for this issue by identifying consistent findings in our population to those previously found in Australia,^10^, ^11^ New Zealand,^12^ and North America.^11^ We recognize that our sample size might represent a limiting factor in our study. Previous reports on this subject had samples that ranged from 67^12^ to 322 patients.^11^ This last one represented a multicenter study that comprised six different institutions. We believe that further studies with such representative samples are still needed. Our results stress this necessity and might encourage larger multicenter cohort studies.

Herein, we emphasize the association of site of metastasis as a prognostic marker, other clinical and pathological features were also identified as associated with a disease-free interval in our sample. This includes the site of the primary tumor, tumor diameter, DOI, invasion beyond subcutaneous fat, neck metastasis at first presentation, TNM stage, and number of positive lymph nodes. It is important to highlight that all our parotid metastases included a solitary node or solitary mass composed of fused nodes not countable. So why do those cases of parotid-only metastases tend to have better outcomes? Is it a site-specific trend or a bias related to a usually single nodular disease? There is insufficient literature to support that a patient with a single parotid metastasis has a different prognosis compared with one with a single neck metastasis. An interesting study by Ebrahimi et al. found both single parotid and neck node presentations associated with favorable outcomes, although the authors did not show an internal comparison between neck and parotid metastasis. We did not find the same better outcomes in this group of solitary parotid metastasis. About 1/3 of this group from our study died by disease, different from the 92 % of 5 year DSS found by Ebrahimi et al.^19^

## Conclusion

In conclusion, our results demonstrated a better survival in patients with isolated involvement of parotid lymph nodes compared with those with neck involvement. We believe that such cases of isolated metastasis in the parotid have an intermediate outcome between non-metastatic cases and those with neck disease. Our findings are in agreement with previous reports conducted in different populations. However, we support a recommendation that further study comprising larger samples need to be performed to confirm if a review of the current staging system is in fact necessary.

## Ethical approval

All procedures performed in studies involving human participants were in accordance with the ethical standards of the institutional and/or national research committee and with the 1964 Helsinki declaration and its later amendments or comparable ethical standards.

## Informed consent

As this study was retrospective and with no intervention, no informed consent was applied.

## Conflicts of interest

The authors declare no conflicts of interest.

## References

[1] Bray F., Ferlay J., Soerjomataram I., Siegel R.L., Torre L.A., Jemal A. (2018). Global cancer statistics 2018: GLOBOCAN estimates of incidence and mortality worldwide for 36 cancers in 185 countries. CA Cancer J Clin..

[2] Global Cancer Observatory. http://gco.iarc.fr/tomorrow/home. Accessed March 3, 2019.

[3] Skulsky S.L., O’Sullivan B., McArdle O., Leader M., Roche M., Conlon P.J. (2017). Review of high-risk features of cutaneous squamous cell carcinoma and discrepancies between the American Joint Committee on Cancer and NCCN Clinical Practice Guidelines In Oncology. Head Neck..

[4] Mourouzis C., Boynton A., Grant J., Umar T., Wilson A., Macpheson D. (2009). Cutaneous head and neck SCCs and risk of nodal metastasis ‒ UK experience. J Craniomaxillofac Surg..

[5] Moore B.A., Weber R.S., Prieto V., El-Naggar A., Holsinger F.C., Zhou X. (2005). Lymph node metastases from cutaneous squamous cell carcinoma of the head and neck. Laryngoscope..

[6] Karia P.S., Morgan F.C., Califano J.A., Schmults C.D. (2018). Comparison of tumor classifications for cutaneous squamous cell carcinoma of the head and neck in the 7th vs. 8th edition of the AJCC Cancer Staging Manual. JAMA Dermatol..

[7] Edge S.B., Compton C.C. (2010). The American Joint Committee on Cancer: the 7^th^ edition of the AJCC cancer staging manual and the future of TNM. Ann Surg Oncol..

[8] Amin M.B., Greene F.L., Edge S.B., Compton C.C., Gershenwald J.E., Brookland R.K. (2017). The eighth edition AJCC Cancer Staging Manual: Continuing to build a bridge from a population-based to a more “personalized” approach to cancer staging. CA Cancer J Clin.

[9] Moeckelmann N., Ebrahimi A., Dirven R., Liu J., Low T.H., Gupta R. (2018). Analysis and comparison of the 8^th^ edition American Joint Committee on Cancer (AJCC) NodalStaging System in cutaneous and oral squamous cell cancer of the head and neck. Ann Surg Oncol.

[10] O’Brien C.J., McNeil E.B., McMahon J.D., Pathak I., Lauer C.S., Jackson M.A. (2002). Significance of clinical stage, extent of surgery, and pathologic findings in metastatic cutaneous squamous carcinoma of the parotid gland. Head Neck..

[11] Andruchow J.L., Veness M.J., Morgan G.J., Gao K., Cliford A., Shannon K.F. (2006). Implications for clinical staging of metastatic cutaneous squamous carcinoma of the head and neck based on a multicenter study of treatment outcomes. Cancer..

[12] Ch’ng S., Maitra A., Lea R., Brasch H., Tan S.T. (2006). Parotid metastasis ‒ An independent prognostic factor for head and neck cutaneous squamous cell carcinoma. J Plast Reconstr Aesthet Surg..

[13] Hirshoren N., Danne J., Dixon B.J., Magarey M., Kleid S., Webb A. (2017). Prognostic markers in metastatic cutaneous squamous cell carcinoma of the head and neck. Head Neck..

[14] Givi B., Andersen P.E., Diggs B.S., Wax M.K., Gross N.D. (2011). Outcome of patients treated surgically for lymph node metastases from cutaneous squamous cell carcinoma of the head and neck. Head Neck..

[15] Bachar G., Mizrachi A., Rabinovics N., Guttman D., Shpitzer T., Ad-El D. (2016). Prognostic factors in metastatic cutaneous squamous cell carcinoma of the head and neck. Ear Nose Throat.

[16] Califano J.A., Lydiatt W.M., Nehal K.S., O’Sullivan B., Schmults C., Seethala R.R., Amin M.B., Edge S.B., Greene F.L. (2017). AJCC Cancer Staging Manual.

[17] Karia P.S., Morgan F.C., Ruiz E.S., Schmults C.D. (2017). Clinical and incidental perineural invasion of cutaneous squamous cell carcinoma: A systematic review and pooled analysis of outcomes data. JAMA Dermatol..

[18] Cheraghlou S., Otremba M., Kuo Yu P., Agogo G.O., Hersey D., Judson B.L. (2018). Prognostic value of lymph node yield and density in head and neck malignancies. Otolaryngol Head Neck Surg..

[19] Ebrahimi A., Clark J.R., Lorincz B.B., Milross C.G., Veness M.J. (2012). Metastatic head and neck cutaneous squamous cell carcinoma: defining a low-risk patient. Head Neck..

